# Factors Associated with the 18-Month Cumulative Incidence of Seroconversion of Active Infection with *Taenia solium* Cysticercosis: A Cohort Study among Residents of 60 Villages in Burkina Faso

**DOI:** 10.4269/ajtmh.18-0294

**Published:** 2018-09-04

**Authors:** Veronique Dermauw, Hélène Carabin, Rasmané Ganaba, Assana Cissé, Zékiba Tarnagda, Sarah Gabriël, Pierre Dorny, Athanase Millogo

**Affiliations:** 1 Department of Biomedical Sciences, Institute of Tropical Medicine, Antwerp, Belgium;; 2 Department of Biostatistics and Epidemiology, College of Public Health, University of Oklahoma Health Sciences Center, Oklahoma City, Oklahoma;; 3 Agence de Formation de Recherche et d’Expertise en Santé pour l’Afrique (AFRICSanté), Bobo Dioulasso, Burkina Faso;; 4 Institut de Recherche en Sciences de la Santé, Bobo Dioulasso, Burkina Faso;; 5 Department of Veterinary Public Health and Food Safety, Faculty of Veterinary Medicine, Ghent University, Merelbeke, Belgium;; 6 Laboratory of Veterinary Parasitology, Faculty of Veterinary Medicine, Ghent University, Merelbeke, Belgium;; 7 Centre Hospitalier Universitaire Souro Sanou, Bobo Dioulasso, Burkina Faso

## Abstract

Taeniasis/cysticercosis (CC) is an important disease complex with significant burden. This large-scale cohort study aimed at estimating and exploring individual- and village-level factors associated with the cumulative incidences of seroconversion (SC) and seroreversion (SR) of active human CC in three provinces of Burkina Faso. In 60 villages, blood samples were collected and interviews regarding sociodemographic variables and knowledge, attitude, and practices toward the disease complex were conducted at baseline and 18-month follow-up (*N* = 2,211), with the presence of active CC being determined using the B158/B60 antigen enzyme-linked immunosorbent assay (Ag-ELISA). The 18-month Ag SC and SR were estimated at 3.3% (95% confidence interval [CI]: 2.6; 4.2%) and 35.8% (95% CI: 24.5; 48.5%), respectively. Marked provincial differences were found for the 18-month Ag SC (Boulkiemde: cumulative incidence ratio [CIR]: 2.41 (95% CI: 1.21; 4.78) and Nayala: CIR: 3.28 (95% CI: 1.37; 7.84), compared with Sanguie), while not being significantly associated with other sociodemographic factors. A continued refraining from pork consumption was associated with a lower 18-month Ag SC (CIR: 0.55 [95% CI: 0.28; 1.07]), whereas at the village level, the percentage of households owning pigs was associated with a higher 18-month Ag SC (CIR: 1.03 [95% CI: 1.01; 1.05]). In conclusion, this is one of few cohort studies and the first to have enough power to assess possible causal links between individual- and village-level variables and CC in humans. Variables linked to province, pig raising, and pork consumption behaviors were found to cause Ag SC in humans. The latter results further support the importance of adopting a One Health approach to the control of CC.

## INTRODUCTION

The zoonotic disease complex *Taenia solium* taeniasis/cysticercosis (CC) causes important monetary and nonmonetary burden in endemic areas^1–5^ as well as in countries where the life cycle is unlikely to be completed, such as the United States.^6^ In its most severe form, *T. solium* cysticerci establish in the brain, causing a condition called neurocysticercosis (NCC), characterized by a range of neurological symptoms and signs, the most common being epilepsy, severe chronic headaches, and focal deficits.^7^ Overall, *T. solium* has been estimated to incur the largest number disability-adjusted life years among foodborne parasitic infections globally.^8^ In most sub-Saharan countries, including Burkina Faso, *T. solium* is endemic in at least some areas.^9^

Most epidemiological studies exploring risk factors for human CC have so far used cross-sectional designs.^9–14^ Although cross-sectional designs are helpful in determining the present distribution and frequency of an outcome in a population, they cannot be used for causal inference unless the exposure of interest does not change through time (i.e., gender). In addition, associations found between exposures and an outcome in cross-sectional studies may actually reflect an association with the duration of the outcome rather than its incidence. In cross-sectional studies on CC, the temporality of the exposure to a risk factor relative to the initial infection is unknown, which can lead to important biases when assessing the role played by risk factors. For example, the infection could have occurred before exposure, leading to the detection of a noncausal association, or one causal factor of the infection could have disappeared by the time of sampling, leading to the nondetection of a true causal association. This temporality problem is often aggravated by the use of antibody (Ab) detecting tests,^12–14^ measuring exposure instead of active infection, measured in an antigen (Ag)-detecting test format.^15^

Despite the important limitations of cross-sectional studies, only three cohort studies have estimated the cumulative incidences of SC and SR of human CC (Table 1). Garcia et al.^16^ reported the SC and SR based on Ab detection in small-scale population longitudinal sero-surveys in Colombia and Peru, whereas Mwape et al.^17^ and Coral-Almeida et al.^18^ described the SC and SR both for Ab and Ag obtained from large-scale cohort studies in Zambia and Ecuador, respectively. In the two latter cohort studies, no clear age- or gender-associated patterns in Ag or Ab SC were found.^17,18^ To our knowledge, no study has explored the association of other factors with SC to human CC.

**Table 1:** Cumulative incidences of SC and SR to human CC reported in literature

	SC			SR			
Country	*n*	Time	SC, %	*n*	Time	SR, %	Reference
Antibody based							
Colombia	NA	NA	NA	23–32	1 year	43–34	Garcia et al.^16^
Peru	145	1 year	25	19	1 year	32	Garcia et al.^16^
	258	3 year	8	140	3 year	49	Garcia et al.^16^
Ecuador	288	6 month	9	135	6 month	19	Coral-Almeida et al.^18^
	226	7 month	7.5	101	7 month	26	Coral-Almeida et al.^18^
	264	13 month	9	120	13 month	28	Coral-Almeida et al.^18^
Zambia	106	6 month	17	55	6 month	35	Mwape et al.^17^
	107	6 month	21	54	6 month	26	Mwape et al.^17^
	106	1 year	24	55	1 year	33	Mwape et al.^17^
Antigen based							
Ecuador	421	6 month	0.0	3	6 month	0.0	Coral-Almeida et al.^18^
	317	7 month	0.3	1	7 month	0.0	Coral-Almeida et al.^18^
	373	13 month	0.5	1	13 month	100	Coral-Almeida et al.^18^
Zambia	758	6 month	7	109	6 month	33	Mwape et al.^17^
	742	6 month	4	125	6 month	38	Mwape et al.^17^
	758	1 year	6	109	1 year	44	Mwape et al.^17^

SC = seroconversion; SR = seroreversion; NA = not available.

The present study aimed, therefore, at estimating the 18-month Ag SC and Ag SR of CC and at identifying risk factors for active CC in 60 villages in three provinces in Burkina Faso.

## MATERIALS AND METHODS

### Ethical clearance.

Ethical approval was obtained from the University of Oklahoma Health Sciences Center Institutional Review Board and the Centre Muraz ethical review panel in Burkina Faso. Consent forms were read and explained to all potential participants (participant, mother/chief of the household, and pig owner) and field staff were present to respond to any questions with regard to the study. Consenting participants signed the consent forms when literate or put a cross when not. For all children younger than 18 years, parents consented, and children older than 10 years were also asked for their assent. A local witness was present during all consents. A bar of soap was offered to each participant as an incentive for their participation.

### Study design.

This cohort study used data from the 18-month pre-randomization period of a cluster-randomized controlled trial (CRCT) aimed at estimating the effectiveness of an educational program to reduce human and porcine CC.^19^

### Setting and participants.

The study was conducted in three provinces of Burkina Faso: Nayala, Boulkiemde, and Sanguie. Reasons for province inclusion and selection procedures for study villages, households, concessions (i.e., a group of households living in a compound), and participants were previously described.^9,19,20^ Briefly, the three provinces were selected based on their large pig population (Boulkiemde and Sanguie) or neighboring location (Nayala). Departments where there was a record of some pig raising were selected (30 of 31 departments in the three provinces) and two villages per department with at least 1,000 inhabitants and pig raising, present on official maps and separated from other study villages by at least 5 km, were randomly selected for future blocked randomization in the CRCT.

In each village, 80 concessions were sampled using a stratified random sampling approach. Ten concessions were first randomly selected among those raising sows, followed by 30 concessions among those raising piglets (with or without sows) and by 40 concessions among others (with or without pigs). One household was randomly selected in each sampled concession and one eligible individual (aged at least 5 years, village resident for at least 1 year, and not planning to move in the following 3 years) randomly selected from each household was asked for his/her consent to participate in the CRCT.

As described elsewhere,^19^ potential participants were first asked if they were willing to provide a blood sample on three occasions over the next 3 years until 60 participants in each village consented to the serological component of the study. Participants refusing to participate to the serological follow-up were included in the general follow-up to measure knowledge attitudes and practices toward *T. solium* and development of epilepsy and severe chronic headaches. Participants confirmed by the study neurologist as having epileptic seizures, epilepsy, or severe chronic headaches at baseline were excluded from all follow-up measurements. The analytical sample of the present study includes data from the baseline visit (February 2011 to January 2012) and the pre-randomization visit taking place 18 months later (August 2012 to July 2013).

### Variable definition and measurement.

#### Outcome.

Consenting participants were interviewed 18-months apart by a field team in each village. A study physician and phlebotomist visited the villages at baseline and follow-up, respectively, to collect a blood sample from the 60 participants having consented to the serological component of the study. The villages were visited in the same order and 18 months apart. Because of unforeseen circumstances (see Carabin et al.^19^ for more details), the phlebotomist was not available when the villages in Nayala were visited and some participants were absent during the initial pre-randomization sampling period. To reduce the number of missing samples, a physician was sent to collect all blood samples in Nayala and to villages with a high number of participants absent during the initial phlebotomist visit. This resulted in a larger sampling interval between baseline and 18-month follow-up in Nayala as compared with other provinces and in longer intervals between sampling for some participants in other provinces.

Blood samples were obtained from the antebrachium vein through venipuncture with syringe and 10 mL Venosafe serum gel tubes. After collection, tubes were transported and stored in a cooler. At the end of each day or the following day, the serum samples were transported to a nearby health facility where they could be stored in a refrigerator. Within 3 days after blood collection, the sera were frozen and stored at −20°C. Every 4–8 weeks, the sera were transported to the Institut de Recherche en Sciences de la Santé, Bobo Dioulasso, and stored there at −20°C until analysis.

As the focus of our study was to specifically investigate the 18-month cumulative incidence of SC and SR of active infection, as opposed to exposure, the latter which is measured by the presence of antibodies,^15^ we opted for an antigen-detecting test format only. The presence of excretory–secretory circulating antigens of the metacestode of *T. solium* was tested in serum samples by means of the B158/B60 enzyme-linked immunosorbent assay (Ag-ELISA).^15,21^ The optical density (OD) of each serum sample was compared with the mean OD of eight reference negative human sera samples at a probability level of *P* = 0.001 to determine the test result.^22^ A sensitivity of 90% (95% Bayesian credible interval [BCI]: 80; 99%) and a specificity of 98% (95% BCI: 97; 99%) for the detection of active infection had been reported for this test in Ecuador.^15^

#### Exposure.

At the baseline, a questionnaire was used to screen study participants for epilepsy and severe chronic headaches as well as to collect data on sociodemographic factors and practices regarding pork consumption, drinking water, sanitation, self-reported tapeworm infection, and knowledge of the life cycle of *T. solium* (see Supplemental Material 1). Furthermore, the chief (i.e., the head) of each participating household was asked about sanitation and drinking water practices and available assets in the household (see Supplemental Material 2). Moreover, the senior woman of each household was asked questions about pork preparation in addition to latrine access and use by household members (see Supplemental Material 3). Finally, in the selected 40 pig-raising concessions, the pig owner was asked to respond to a questionnaire regarding pig management and knowledge of porcine CC (see Supplemental Material 4). Although the same questionnaire was used at the baseline and pre-randomization for the chief, senior woman of the household, and pig owners, a shorter questionnaire interview was used for each participant at the 18-month follow-up, measuring practices with regard to pork consumption, drinking water, sanitation, and self-reported tapeworm infection as well as knowledge of the life cycle of *T. solium* (see Supplemental Material 5). Finally, soil samples were obtained in each village (between March and November 2014), and the percentage of sand, silt, and clay as well as pH were measured as described earlier.^9^

### Data management and statistical analyses.

#### Data management.

**Figure 1.**
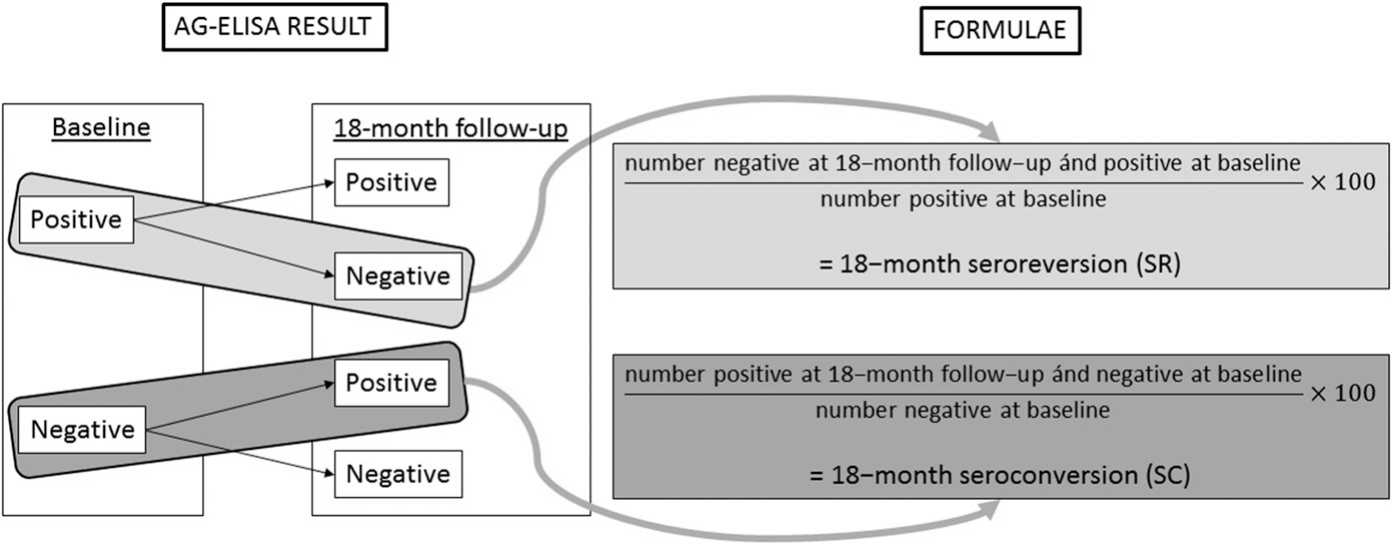
Flow chart: calculation of the 18-month seroconversion (SC) and seroreversion (SR).

All data were recorded on personal digital assistants programmed to generate an Access database. The 18-month SC was defined as the number of study participants being Ag-ELISA negative at the baseline and positive at the pre-randomization visit, divided by the number of participants being Ag-ELISA negative at the baseline. The 18-month SR was defined as the number of participants being Ag-ELISA positive at the baseline and negative at the pre-randomization visit, divided by the number of participants being Ag-ELISA positive at the baseline (Figure 1).

Changes between the baseline and the 18-month follow-up responses to the questionnaire were evaluated and categorized into the following: “improved response,” “deteriorated response,” “unchanged, good response,” or “unchanged, bad response.” An “improved response” was defined as an improvement in knowledge about the life cycle of *T. solium* or having a behavioral change from risky to protective in terms of risk of CC from the baseline to the pre-randomization visit. A “deteriorated response” was defined as losing knowledge about the life cycle or going from a protective behavior to a risky one during that period. An “unchanged, good response” was defined as having a response at both visits, reflecting life cycle knowledge or protective behavior. An “unchanged, bad response” was defined as having a response at both visits, reflecting an absence of knowledge about the life cycle or constant risky behavior.

A selection of variables was also expressed at the village level as the percentage of participants/household heads responding positively to a question or belonging to a certain category (e.g., percentage of participants who reported ever having had a tapeworm and percentage of households with wealth quintile of four or five).

#### Statistical analyses.

The differences (and 95% confidence intervals [CIs]) in sociodemographic characteristics between eligible individuals with a sample at the baseline only and those being sampled at both visits were estimated using the command “prop.test” (“stats” package). The cumulative incidence of SC and SR and related 95% CIs were calculated using the “binom.test” command (“epitools” package).

The association of the 18-month SC with potential risk factors was investigated using generalized linear mixed models with a binomial family and log link (i.e., log-binomial models), with the type of concession and sampling interval inserted as fixed effects and the village as random effect (command “glmer,” package “lme4”). The effect of each variable of interest on the SC was first explored using a random-effect log-binomial model with village as a random effect and type of concession and sampling interval as fixed effects. Variables showing a *P* value < 0.10 in these models were subsequently inserted in a multivariable random-effect log-binomial model with village as a random effect and type of concession and sampling interval as fixed effects. Province, age, and gender were added as fixed effects to the multivariable models. Three multivariable models were run, one with individual-level variables and with individual- and village-level variables, and the last one including both as well as soil variables. The model fit was evaluated based on the Akaike information criterion. The cumulative incidence ratios (CIRs) of SC for the fixed effects in the models and their 95% Wald CIs were calculated using the “confint.merMod” command (package “lme4”). Because of the low number of cases exhibiting SR, this outcome parameter was not modeled. Variables with 95% CI excluding one were considered as statistically significant. All data were coded in Stata 13 and analyzed in R version 3.4.3 (StataCorp., College Station, TX).^23^

## RESULTS

### Participants and descriptive data.

The analytical sample consisted of 2,211 individuals providing blood at both the baseline and pre-randomization visits (median: 39 participants/village, range: 8–53), among the 3,554 eligible individuals providing a serological sample at the baseline. This loss of follow-up resulted from unexpected population migration in large part due to a new gold rush in the study areas, short-term absenteeism associated with social events, and market activities. The proportion of participation to the pre-randomization sampling differed between provinces and age groups (Table 2). Female participants, those who had ever owned pigs, belonged to a concession owning sows, or had ever heard that their pigs were infected with cysticerci, were more likely to participate. Participants being Ag-ELISA positive at the baseline were equally likely to participate as negative individuals.

**Table 2:** Comparison of sociodemographic characteristics of 3,554 individuals eligible for follow-up consenting to the serological component of a study conducted in 60 villages of Burkina Faso, who did (*n* = 2,211) and did not (*n* = 1,343) have samples obtained both at the baseline and pre-randomization 18-month follow-up visits

		Both sera			Difference	
Variable	Categories	No	Yes		% (95% CI)	
Ag-ELISA	Positive	42	67	(61.5%)	−0.8%	(−10.0; 8.5%)
	Negative	1,301	2,144	(62.2%)	–	–
Province (0 missing)	Boulkiemde	655	1,128	(63.3%)	–	–
	Nayala	147	434	(74.7%)	11.4%	(7.3; 15.6%)*
	Sanguie	541	649	(54.5%)	−8.7%	(−12.3;−5.1%)*
Age (years) (42 missing)	6–17	469	700	(59.9%)	–	–
	18–30	308	359	(53.8%)	−6.1%	(−10.8;−1.3%)*
	31–40	176	371	(67.8%)	7.9%	(3.1; 12.8%)*
	> 40	366	763	(67.6%)	7.7%	(3.8; 11.6%)*
Gender (28 missing)	Female	692	1,232	(64.0%)	–	–
	Male	635	967	(60.4%)	−3.7%	(−6.9; 0.5%)*
School attendance (30 missing)	No	918	1,532	(62.5%)	–	–
	Yes	408	666	(62.0%)	−0.5%	(−4.0; 3.0%)
Ever had pigs (31 missing)	No	935	1,414	(60.2%)	–	–
	Yes	390	784	(66.8%)	6.6%	(3.2; 9.9%)*
Eating pork now (32 missing)	No	423	660	(60.9%)	–	–
	Yes	901	1,538	(63.1%)	2.1%	(−1.4; 5.6%)
Pork eating history (31 missing)	Never	333	510	(60.5%)	–	–
	Now	901	1,538	(63.1%)	2.6%	(−1.3; 6.4%)
	In the past	91	150	(62.2%)	1.7%	(−5.2; 8.7%)
Concession type (0 missing)	Sow	155	331	(68.1%)	–	–
	Piglet	492	850	(63.3%)	−4.8%	(−9.6; 0.1%)
	Any	696	1,030	(59.7%)	−8.4%	(−13.2;−3.7%)*
HH owns pigs (32 missing)	No	370	558	(60.1%)	–	–
	Yes	955	1,639	(63.2%)	3.1%	(−0.6; 6.7%)
Where pork is eaten (0 missing	At home	429	806	(65.3%)	–	–
	Other concession	161	261	(61.8%)	−3.4%	(−8.8; 1.9%)
	Village market	225	353	(61.1%)	−4.2%	(−9.0; 0.6%)
	Other village market	86	118	(57.8%)	−7.4%	(−14.7;−0.1%)
Told pigs had CC (0 missing) (among those with pigs)	No	349	667	(65.6%)	–	–
	Yes	41	117	(74.1%)	8.4%	(1.0; 15.8%)*
Use toilet to defecate (31 missing)	No	1,166	1,899	(62.0%)	–	–
	Yes	159	299	(65.3%)	3.3%	(−1.4; 8.0%)
Access to a latrine (42 missing)	No	1,171	1,911	(62.0%)	–	–
	Yes	149	281	(65.3%)	3.3%	(−1.5; 8.2%)
HH has a latrine (6 missing)	No	1,170	1,897	(61.9%)	–	–
	Yes	167	314	(65.3%)	3.4%	(−1.2; 8.0%)
Heard about tapeworm (31 missing)	No	516	834	(61.8%)	–	–
	Yes, did not have it	689	1,150	(62.5%)	0.8%	(−2.7; 4.2%)
	Yes, had it	120	214	(64.1%)	2.3%	(−3.5; 8.1%)
Wealth quintile (3 missing)	0	268	416	(60.8%)	–	–
	1	263	454	(63.3%)	2.5%	(−2.6; 7.6%)
	2	275	439	(61.5%)	0.7%	(−4.4; 5.8%)
	3	280	433	(60.7%)	−0.1%	(−5.2; 5.0%)
	4	254	469	(64.9%)	4.0%	(−1.0; 9.1%)
Occupation (30 missing)	Student/pupil	292	508	(63.5%)	–	–
	Farmer	505	798	(61.2%)	−2.3%	(−6.5; 2.0%)
	Housewife/cleaner	451	794	(63.8%)	0.3%	(−4.0; 4.5%)
	Salaried/commerce/unemployed	78	98	(55.7%)	−7.8%	(−15.9; 0.2%)

CC = cysticercosis; HH = household; 95% CI = 95% confidence interval for the difference in proportions.

* *P* < 0.05.

### Outcome data.

Of the 2,211 study participants, 3.0% (95% CI: 2.4; 3.8%) were positive for active CC at the baseline, whereas 5.2% (95% CI: 4.3; 6.2%) were positive at the 18-month follow-up visit (Table 3). The overall cumulative incidence of Ag SC was 3.3% (95% CI: 2.6; 4.2%), whereas the overall cumulative incidence of Ag SR was 35.8% (95% CI: 24.5; 48.5%).

**Table 3:** Prevalence of and cumulative incidences of SC and SR to active CC in 2,211 individuals eligible for follow-up consenting to the serological component of a study conducted in 60 villages of Burkina Faso, who had samples both at the baseline and pre-randomization 18-month follow-up visits

Parameter	Province	Total	*n*	% (95% CI)
Prevalence (baseline)	Boulkiemde	1,128	48	4.3 (3.2; 5.6)
	Nayala	434	12	2.8 (1.6; 4.8)
	Sanguie	349	7	1.1 (0.01; 2.2)
	Total	2,211	67	3.0 (2.4; 3.8)
Prevalence (18 month follow-up)	Boulkiemde	1,128	76	6.7 (5.4; 8.4)
	Nayala	434	20	4.6 (3.0; 7.0)
	Sanguie	349	18	2.8 (1.8; 4.4)
	Total	2,211	114	5.2 (4.3; 6.2)
SC	Boulkiemde	1,080	43	4.0 (2.9; 5.3)
	Nayala	422	14	3.3 (1.8; 5.5)
	Sanguie	642	14	2.2 (1.2; 3.6)
	Total	2,144	71	3.3 (2.6; 4.2)
SR	Boulkiemde	48	15	31.3 (18.7; 46.3)
	Nayala	12	6	50.0 (21.1; 78.9)
	Sanguie	7	3	42.9 (9.9; 81.6)
	Total	67	24	35.8 (24.5; 48.5)

SC = seroconversion; SR = seroreversion; 95% CI = 95% binomial exact confidence interval.

### Univariate analyses.

In models investigating the effect of sociodemographic variables (Table 4), a significant difference in 18-month Ag SC was found between participants from Boulkiemde in comparison to those from Sanguie. No significant age or gender differences were found, yet a nonsignificant difference in SC was found for participants older than 40 years, compared with those between 6 and 17 years old (CIR: 1.79 [95% CI: 0.97; 3.32]). Farmers and housewives had an insignificantly higher 18-month Ag SC compared with students (CIR: 1.91 [95% CI: 0.91; 4.04], CIR: 1.93 [95% CI: 0.92; 4.07], respectively) and those attending school an insignificantly lower 18-month Ag SC versus those who did not (CIR: 0.58 [95% CI: 0.32; 1.06]). No differences in 18-month Ag SC could be detected between wealth quintiles, nor for any of the other socioeconomic characteristics of the study population.

**Table 4:** Association between individual-level sociodemographic factors and the cumulative incidence of SC among 2,211 individuals providing both serum at the baseline and pre-randomization 18-month follow-up visits in 60 villages of Burkina Faso

		SC		
Variable		Total	*n*, SC	CIR (95% CI)
Province	Boulkiemde	1,037	43 (4.0%)	2.27 (1.11; 4.66)*
	Nayala	408	14 (3.3%)	1.71 (0.72; 4.08)
	Sanguie	628	14 (2.2%)	Ref
Age (years)	6–17	673	17 (2.5%)	Ref
	18–30	340	14 (4.0%)	1.73 (0.84; 3.58)
	31–40	347	9 (2.5%)	1.09 (0.47; 2.55)
	> 40	695	31 (4.3%)	1.79 (0.97; 3.32)†
Gender	Male	881	29 (3.2%)	1.00 (0.61; 1.63)
	Female	1,180	42 (3.4%)	Ref
Wealth quintile	0	381	16 (4.0%)	1.20 (0.55; 2.64)
	1	418	18 (4.1%)	1.44 (0.70; 2.94)
	2	417	9 (2.1%)	0.70 (0.29; 1.69)
	3	406	15 (3.6%)	1.33 (0.64; 2.77)
	4	448	13 (2.8%)	Ref
Occupation	Student/pupil	491	10 (2.0%)	Ref
	Farmer	730	28 (3.7%)	1.91 (0.91; 4.04)†
	Housewife	745	30 (3.9%)	1.93 (0.92; 4.07)†
	Others	94	3 (3.1%)	1.66 (0.46; 5.99)
School attendance	Yes	634	15 (2.3%)	0.58 (0.32; 1.06)†
	No	1,426	56 (3.8%)	Ref

CC = cysticercosis; CIR = cumulative incidence ratio; Ref = reference; SC = seroconversion; 95% CI = 95% Wald confidence interval for fixed effects in mixed models with village as random variable and type of concession, sampling interval, and the variable of interest as fixed effects.

* *P* < 0.05.

† *P* < 0.10.

For those variables measuring practices and knowledge toward taeniasis/cysticerocis, current and past pork-eating behaviors were significantly associated with 18-month Ag SC (CIR: 2.75 [95% CI: 1.28; 5.89], CIR: 3.85 [95% CI: 1.46; 10.10], respectively) (Table 5). Those eating pork at home (CIR: 2.49 [95% CI: 1.11; 5.63]), but especially those eating pork at village markets (own village market: CIR: 3.71 [95% CI: 1.56; 8.84], other village market: CIR: 4.50 [95% CI: 1.48; 13.71]), had a higher 18-month Ag SC versus those who never ate pork. In that comparison, participants having eaten pork before also had a higher 18-month Ag SC compared with those who never ate pork (CIR: 3.88 [95% CI: 1.48; 10.20]). Those consuming non–oven-baked pork also had an insignificantly higher 18-month Ag SC versus those who did not report eating pork (CIR: 1.66 [95% CI: 0.92; 3.00]). Having heard about porcine CC was related with a higher 18-month Ag SC (CIR: 1.81 [95% CI: 1.04; 3.14]) (Table 6).

**Table 5:** Association between individual-level practices and the cumulative incidence of seroconversion among 2,211 individuals providing both serum at the baseline and pre-randomization 18 month follow-up visits in 60 villages of Burkina Faso

		SC		
Variable		Total	*n*, SC	CIR (95% CI)
Pork consumption	Eats pork now	1,426	53 (3.6%)	2.75 (1.28; 5.89)*
	Ate pork in the past	137	8 (5.5%)	3.85 (1.46; 10.10)*
	Never ate pork	497	10 (2.0%)	Ref
Eating oven-baked pork	Eats oven baked pork	36	2 (5.3%)	2.68 (0.64; 11.34)
	Eats other type of pork	1,390	51 (3.5%)	1.66 (0.92; 3.00)†
	Never ate pork	633	18 (2.8%)	Ref
Location pork eating	Eats pork at home only	754	25 (3.2%)	2.50 (1.11; 5.63)‡
	Eats pork in other concession	250	7 (2.7%)	1.67 (0.54; 5.11)
	Eats pork at the village market	321	16 (4.7%)	3.71 (1.56; 8.84)*
	Eats pork in other village market	101	5 (4.7%)	4.50 (1.48; 13.71)*
	Ate pork before, not anymore	137	8 (5.5%)	3.88 (1.48; 10.20)*
	Never ate pork	496	10 (2.0%)	Ref
Self-reported toilet use	Yes	285	10 (3.4%)	1.04 (0.53; 2.07)
	No	1,775	61 (3.3%)	Ref
Mother reports HH access latrine	Yes	268	10 (3.6%)	1.07 (0.54; 2.15)
	No	1,786	61 (3.3%)	Ref
Chief reports HH has latrine	Yes	302	8 (2.6%)	0.71 (0.33; 1.51)
	No	1,771	63 (3.4%)	Ref
Ever had pigs	Yes	729	29 (3.8%)	1.48 (0.90; 2.41)
	No	1,331	42 (3.1%)	Ref
Told pigs had CC^1^	Yes	105	6 (5.4%)	1.56 (0.65; 3.77)
	No	624	23 (3.6%)	Ref

CC = cysticercosis; CIR = cumulative incidence ratio; HH = household; Ref = reference; SC = seroconversion; 95% CI = 95% Wald confidence interval for fixed effects in mixed models with village as random variable and type of concession, sampling interval, and the variable of interest as fixed effects.

* *P* < 0.01.

† *P* < 0.10.

‡ *P* < 0.05.

**Table 6:** Association between individual-level knowledge and the cumulative incidence of SC among 2,211 individuals providing both serum at the baseline and pre-randomization 18-month follow-up visits in 60 villages of Burkina Faso

		SC		
Variable		Total	*n*, SC	CIR (95% CI)
Has heard about porcine CC	Yes	1,290	51 (3.8%)	1.81 (1.04; 3.14)*
	No	770	20 (2.5%)	Ref
Knows where to find cysts in a live pig (under the tongue)	Yes	1,006	45 (4.3%)	2.18 (0.86; 5.52)
	No	243	5 (2.0%)	Ref
Knows how a pig acquires CC (eating human feces)	Yes	68	3 (4.2%)	1.28 (0.40; 4.13)
	No	1,181	47 (3.8%)	Ref
Knows how to recognize a tapeworm infection (see worm in feces)	Yes	606	19 (3.0%)	0.71 (0.39; 1.30)
	No	620	25 (3.9%)	Ref
Knows how humans contract a tapeworm (eating undercooked pork)	Yes	49	2 (3.9%)	1.11 (0.27; 4.54)
	No	1,177	42 (3.4%)	Ref
Tapeworm knowledge/infection	Had it	193	7 (3.5%)	1.22 (0.72; 2.05)
	Heard about, never had it	1,071	38 (3.4%)	1.34 (0.58; 3.11)
	Does not know it	796	26 (3.2%)	Ref

CC = cysticercosis; CIR = cumulative incidence ratio; Ref = reference; SC = seroconversion; 95% CI = 95% Wald confidence interval for fixed effects in mixed models with village as random variable and type of concession, sampling interval, and the variable of interest as fixed effects.

* *P* < 0.05.

On exploration of 18-month changes in practices and knowledge toward taeniasis/CC (Table 7), a lower 18-month Ag SC was observed for participants who continued to refrain from pork consumption between the baseline and follow-up visit (CIR: 0.46 [95% CI: 0.24; 0.89]) versus those who continued to consume pork. Participants who continued to refrain from pig production also had a lower 18-month Ag SC (CIR: 0.41 [95% CI: 0.22; 0.79]), versus those who continued to keep pigs.

**Table 7:** Association between individual-level changes in practices and knowledge, and the cumulative incidence of seroconversion among 2,211 individuals providing both serum at the baseline and pre-randomization 18-month follow-up visits in 60 villages of Burkina Faso

		SC		
Variable		Total	*n*, SC	CIR (95% CI)
Change, eating pork	Change, improved	231	5 (2.1%)	0.57 (0.23; 1.43)
	Change, deteriorated	57	3 (5.0%)	1.19 (0.38; 3.75)
	No change, kept good	567	14 (2.4%)	0.46 (0.24; 0.89)†
	No change, kept bad	1,170	47 (3.9%)	Ref
Change, location eating pork	Change, improved	264	13 (4.7%)	1.06 (0.35; 3.20)
	Change, deteriorated	46	2 (4.2%)	1.00 (0.19; 5.26)
	No change, kept good	759	28 (3.6%)	0.77 (0.27; 2.18)
	No change, kept bad	98	4 (3.9%)	Ref
Change, use toilet	Change, improved	271	7 (2.5%)	0.54 (0.22; 1.35)
	Change, deteriorated	74	2 (2.6%)	0.83 (0.20; 3.36)
	No change, kept good	201	7 (3.4%)	0.91 (0.40; 2.04)
	No change, kept bad	1,474	53 (3.5%)	Ref
Change, having pigs	Change, improved	154	5 (3.1%)	0.74 (0.29; 1.91)
	Change, deteriorated	505	19 (3.6%)	0.91 (0.50; 1.64)
	No change, kept good	809	21 (2.5%)	0.41 (0.22; 0.79)‡
	No change, kept bad	557	24 (4.1%)	Ref
Change, knowledge on where to find cyst in live pig (under the tongue)	Change, improved	121	3 (2.4%)	1.59 (0.17; 15.0)
	Change, deteriorated	96	5 (5.0%)	3.71 (0.44; 31.21)
	No change, kept good	774	36 (4.4%)	2.86 (0.39; 20.89)
	No change, kept bad	58	1 (1.7%)	Ref
Change, knowledge on how pig acquires CC (eating human feces)*	Change, improved	24	1 (4.0%)	0.98 (0.14; 6.88)
	Change, deteriorated	55	3 (5.2%)	1.27 (0.41; 3.99)
	No change, kept good	2	0 (0.0%)	–
	No change, kept bad	968	41 (4.1%)	Ref
Change, knowledge on how to recognize tapeworm infection (see worm in feces)*	Change, improved	1	0 (0.0%)	–
	Change, deteriorated	500	15 (2.9%)	0.81 (0.42; 1.57)
	No change, kept good	1	0 (0.0%)	–
	No change, kept bad	508	19 (3.6%)	Ref
Change, knowledge on how humans contract a tapeworm (eating undercooked pork)*	Change, improved	16	0 (0.0%)	–
	Change, deteriorated	38	2 (5.0%)	1.54 (0.38; 6.22)
	No change, kept good	–	–	–
	No change, kept bad	956	32 (3.2%)	Ref

CC = cysticercosis; CIR = cumulative incidence ratio; Ref = reference; SC = seroconversion; 95% CI = 95% Wald confidence interval for fixed effects in mixed models with village as random variable and type of concession, sampling interval and the variable of interest as fixed effects.

* Because of incomplete classes, or too many missing values for these variables, no mixed models were run for these variables, CIR with 95% CI were provided for complete classes only.

† *P* < 0.05.

‡ *P* < 0.01.

Some village-level variables were associated with SC (Table 8). The percentage of households owning pigs (CIR: 1.02 [95% CI: 1.01; 1.04]) and the percentage of households with wealth quintiles four or five (CIR: 1.02 [95% CI: 1.00; 1.04]) were associated with an increasing 18-month Ag SC. The percentage of sand in the village soil was also, yet insignificantly, associated with an increasing 18-month Ag SC (CIR: 1.02 [95% CI: 1.00; 1.04]).

**Table 8:** Associations between village-level factors and the cumulative incidence of SC among 2,211 individuals providing both serum at the baseline and pre-randomization 18-month follow-up visits in 60 villages of Burkina Faso

Variable	CIR (95% CI)
Percentage of participants who reported ever having had a tapeworm	1.04 (0.99; 1.09)
Percentage of participants who reported ever heard about tapeworm, but never had one	1.02 (0.99; 1.05)
Percentage of pigs roaming or tethered during the rainy season and roaming during the dry season	1.00 (0.99; 1.02)
Percentage of households practicing home slaughtering	1.01 (0.99; 1.04)
Percentage of households with home slaughtering for which meat inspection is practiced	1.00 (0.98; 1.02)
Percentage of households owning pigs	1.02 (1.01; 1.04)*
Percentage self-reporting using latrines to defecate	1.00 (0.98; 1.02)
Percentage of households in which mothers declared that family members had access to a latrine	1.00 (0.98; 1.02)
Percentage with wealth quintile of four or five	1.02 (1.00; 1.04)†
Percentage of participants declaring eating pork	1.00 (0.99; 1.02)
Percentage of participants declaring eating pork only at someone’s household (including own)	1.00 (0.98; 1.02)
Percentage of participants declaring eating pork at the market (village market or other)	1.01 (0.99; 1.04)
pH level in soil	1.18 (0.80; 1.73)
Percentage of silt in soil	0.99 (0.96; 1.01)
Percentage of sand in soil	1.02 (1.00; 1.04)‡
Percentage of clay in soil	0.98 (0.94; 1.01)

CIR = cumulative incidence ratio; SC = seroconversion; 95% CI = 95% Wald confidence interval for fixed effects in mixed models with village as random effect and type of concession and the variable of interest as fixed effects.

* *P* < 0.01.

† *P* < 0.05.

‡ *P* < 0.10.

### Multivariable analyses.

In the best fit model for the multivariable analysis including only individual-level variables (Model 1) (Table 9), participants from Boulkiemde had a significantly higher Ag SC than those from Sanguie (CIR: 2.19 [95% CI: 1.08; 4.45]). No differences were found for gender, whereas an insignificantly higher 18-month Ag SC was observed for participants older than 40 years, compared with those between 6 and 17 years old (CIR: 1.71 [95% CI: 0.91; 3.20]). Those who kept refraining from pork consumption had a significantly lower Ag SC than those maintaining pork consumption (CIR: 0.42 [95% CI: 0.21; 0.81]).

**Table 9:** Multivariable associations between individual- and village-level factors and the cumulative incidence of SC among 2,211 individuals providing both serum at baseline and pre-randomization 18-month follow-up visits in 60 villages of Burkina Faso

		CIR (95% CI)	
Variable		Model 1	Model 2
Province	Boulkiemde	2.19 (1.08; 4.45)*	2.41 (1.21; 4.78)*
	Nayala	1.81 (0.76; 4.29)	3.28 (1.37; 7.84)†
	Sanguie	Ref	Ref
Age (years)	6–17	Ref	Ref
	18–30	1.73 (0.82; 3.63)	1.70 (0.81; 3.56)
	31–40	1.09 (0.46; 2.55)	1.12 (0.48; 2.63)
	> 40	1.71 (0.91; 3.20)‡	1.70 (0.91; 3.18)‡
Gender	Male	0.97 (0.58; 1.61)	0.99 (0.60; 1.64)
	Female	Ref	Ref
Change eating pork	Change, improved	0.57 (0.23; 1.44)	0.59 (0.24; 1.47)
	Change, deteriorated	1.08 (0.34; 3.41)	1.10 (0.35; 3.42)
	No change, kept good	0.42 (0.21; 0.81)*	0.55 (0.28; 1.07)‡
	No change, kept bad	Ref	Ref
Percentage household owning pigs	Per unit increase	–	1.03 (1.01; 1.05)†

CIR = cumulative incidence ratio; Ref = reference; SC = seroconversion; 95% CI = 95% Wald confidence interval for fixed effects in mixed models with village as random effect, and type of concession, the sampling interval, and the variables of interest as fixed effect. All models also included province, age, and gender as fixed effects.

Model 1: without village-level variables; Model 2: with village-level variables.

* *P* < 0.05.

† *P* < 0.01.

‡ *P* < 0.10.

In the multivariable model including both individual- and village-level variables (Model 2) (Table 9), the village percentage of pig ownership was associated with an increasing 18-month Ag SC (CIR: 1.03 [95% CI: 1.01; 1.05]). Those who kept refraining from pork consumption had an insignificantly lower Ag SC than those maintaining pork consumption (CIR: 0.55 [95% CI: 0.28; 1.07]). In this model, both participants from Boulkiemde and Nayala had a higher 18-month Ag SC compared with those from Sanguie (CIR: 2.41 [95% CI: 1.21; 4.78], CIR: 3.28 [95% CI: 1.37; 7.84], respectively). Again, no differences were observed between male and female participants, whereas an insignificantly higher 18-month Ag SC was observed for participants older than 40 years, compared with those between 6 and 17 years old (CIR: 1.70 [95% CI: 0.91; 3.18]). In the model including individual-level, village-level variables, and village soil characteristics, the soil variables were not retained in the final model; hence, the best fit model remained Model 2.

## DISCUSSION

This is the first study to pursue an in-depth exploration of risk factors for incidence of human CC. The diagnostic tool used in the present study, the Ag-ELISA, detects circulating antigens of *T. solium*, indicating the presence of an active CC infection.^15^ The 18-month Ag SC in this study was found to be 3.3%, thus suggesting that 3.3% of the study participants negative at the baseline seroconverted, that is, became test positive, and thus developed active CC over the 18-month study period. This value for the 18-month Ag SC is lower than the one found in the cohort study performed in Zambia (12-month Ag SC, 6%),^17^ whereas much higher than that is observed in a cohort study conducted in Ecuador (13-month Ag SC, 0.5%).^18^

In this study, a high percentage (35.8%) of test-positive study participants at the baseline seroreverted, that is, became test negative, over the 18-month study period (the 18-month Ag SR). Seroreversion could indicate that in these study participants positive at the baseline, the present cysticerci calcified and were thus no longer viable (and detectable), yet the participants remained infected and at risk to develop symptomatic NCC. Alternatively, the infection could have been self-cured, a possible hypothesis suggested to explain the presence of transient antibodies in disease-endemic areas in Peru and Colombia.^16^ Overall, the observed value for the 18-month Ag SR (35.8%) was slightly lower than the SR found in the cohort study performed in Zambia (12-month Ag SR, 44%), where the study group at risk (positive at baseline) for SR was larger than that in our study, because of the higher prevalence of active CC (12.5%).^17^ The infection dynamics may have been slower overall in our study population than in the Zambian one; however, the smaller number of seropositive participants at the baseline in our study also introduced more uncertainty into our estimates. In the cohort study in Ecuador, the study group at risk for SR consisted of only one person (positive at the baseline), who did serorevert during the 13-month study period.^18^

In the models investigating the effect of each variable of interest separately, we found the province of residence, pork consumption behavioral, and knowledge of pig CC to be risk factors for 18-month Ag SC, whereas no associations were found for other sociodemographic factors. By contrast, continued refraining from pork consumption and from raising pigs was associated with a lower 18-month Ag SC. At the village level, the percentage of households owning pigs, as well as those with wealth quintile four or five, was associated with a higher 18-month Ag SC.

In the multivariable models, both the provincial differences and the impact of a continued refraining from pork consumption and the percentage of households owning pigs were confirmed. Previous cohort studies investigating the cumulative incidence of SC of human CC could not identify significant differences for age categories or gender^17,18^; other factors have never been investigated before. As in the two previous cohort studies, gender was not found to be a risk factor in our study. This is in contrast to our cross-sectional findings using the baseline data, where males were found to have higher seroprevalences of active CC than females.^9^ One possible explanation for this observation would be that males stay infected for longer than females, which would result in associations with prevalence measures but not with incidence measures. Indeed, females tended to have higher 18-months cumulative incidence of SR than males, although this was not statistically significant because of the small number of individuals seropositive at the baseline and providing samples at both visits (45.5% in females versus 31.3% in males). Again, as in the two previous cohort studies, age category was not found to be a risk factor in our study, whereas in our cross-sectional study, a province by age interaction was observed.^9^ In the present study, the continued refraining from pork consumption was found to be associated with the 18-month Ag SC, an effect which is challenging to explain because it is directly associated with taeniasis, not human CC. Indeed, consumption of undercooked pork is an essential factor for the continuation of the natural life cycle of *T. solium*, with humans serving as definite hosts (i.e., taeniasis).^24^ As we had previously demonstrated a high prevalence of active CC in pigs with estimates of 32.5% and 39.6% in two pilot villages located in the same area,^25^ transmission is thought be widespread. However, the direct role of pork consumption in the acquisition of human CC (with humans then serving as accidental intermediate host) remains unclear. People with taeniasis may in turn cause CC in other humans or themselves through hands contaminated with tapeworm eggs, followed by hand–mouth contact or by ingestion of food handled by a tapeworm carrier (fecal–oral transmission).^26,27^ Another, probably less common, pathway through which individuals can acquire CC is through autoinfection, that is, through reverse peristaltic movements of the intestine.^28,29^ Overall, the observed protective effect could be explained by the fact that people who continuously refrain from eating pork either come from a household or concession where no one consumes pork, leading to the reduction of taeniasis cases and, hence, direct or indirect transmission to others, including the participant, or that it reduces autoinfection in the participating subjects. In our cross-sectional study, a history of pork consumption was equally linked to active CC.^9^ More large-scale cohort studies, including in-depth explorations of within household and concession pork consumption behaviors, are needed to unravel this association.

The percentage of households raising pigs at the village level was an important confounder of the effect that living in Nayala had on 18 months SC. After adjustment, living in Nayala had a stronger impact on SC than living in Boulkiemde as compared with living in Sanguie. The confounding effect of pig raising at the village level is not surprising because Nayala was the province where less households raised pigs. Overall, the effect of the province on SC will need more investigation. There may be unmeasured village-level or province-level contextual or environment factors explaining the differences. For example, the physical environment such as vegetation, humidity, and temperature may be different enough among provinces to impact the survival of parasitic eggs in the environment. People in the different provinces may also have different food or hand hygiene behaviors, not measured here, putting them at higher risk of infection. Variation in the effectiveness of the intervention between provinces was also observed in the CRCT, suggesting that these areas are likely to have contextual factors impacting the epidemiology of CC.^19^

Our study had several limitations. First, various events (e.g., gold mining) caused a reduction in sample size, that is, a lower number of participants with blood samples at the baseline and follow-up than anticipated. Differences in sociodemographic characteristics were also identified for participants who did and did not have samples obtained both at the baseline and pre-randomization 18-month follow-up visits, most relevant of which were adjusted for in the multivariable models. In addition, the seroprevalence of infection was not different between those providing both samples from those with only a sample at the baseline, reducing the potential impact of selection bias on our results. Second, participants in Nayala had a larger sampling interval than those from the other two provinces, yet this was also adjusted for in the multivariable models. Finally, too few cases of 18-month Ag SR were present to allow modeling.

In conclusion, this study is the first to evaluate the association between a range of individual- and village-level variables and the 18-month Ag SC. It provides evidence that continued refraining from pork consumption and village level of pig-keeping as well as contextual characteristics of provinces may influence the occurrence of human CC.

## Supplementary Material

Supplemental materials
