# Supplementary material for: Factors Associated with the 18-Month Cumulative Incidence of Seroconversion of Active Infection with Taenia solium Cysticercosis: A Cohort Study among Residents of 60 Villages in Burkina Faso
Source: Am J Trop Med Hyg. 2018 Sep 4;99(4):1018–27. doi: 10.4269/ajtmh.18-0294 (PMC6159582; doi:10.4269/ajtmh.18-0294)
Supplement: Supplementary file 1 [file tpmd180294.SD1.pdf]

ÉFÉ CAB

Improving pig management to prevent epilepsy in Burkina Faso

Centre Hospitalier Universitaire Souro Sanou, AFRICSanté & University of Oklahoma Health Sciences Center

SCREENING QUESTIONNAIRE

Last name : ________________________ First name : ______________________________

Questionnaire number _____________________

Identification number |__|__|__|__|__|__|__|__|__|__|__|__|

Village ___________________________________

Concession ___________________________________

Household number ________________________

How long have you lived in this village?____(yrs.)

Do you benefit from a health insurance? ☐ Yes ☐ No

If yes, what type of health insurance is it?

☐ Private Health insurance ☐ Mutual Health insurance ☐ Other [*specify*] ________

1 How old are you? _____________ (years)

2 What is your date of birth?____ Day _____ Month _______ Year

3 Sex ☐ Male ☐ Female

4. Did you go to a modern school? ☐ Yes ☐ No [*please go to Q6*]

5. What is the last grade you attended? ___________________________

5.1 What is the highest schooling grade you have completed?

☐ None ☐ CEPE ☐ BEPC ☐ BEP/CAP

☐ Baccalauréat ☐ University degree

6 What is your usual occupation, in other word, what work do you do most of the time [*housewife is an occupation*]?

☐ Farmer ☐ Small business ☐ Handicraft

☐ Salaried (specify) _______________ ☐ Housewife

☐ Other (specify) ______________________

6.1 If you have a usual occupation, what is your monthly salary? ___________________ CFA

7 How many days of work have you missed because of illness in the past month? ______ days

7.1 If you do not have an official employment, how many days have you been unable to attend to your daily chores in the past month (past 30 days)? _____ days

7.2 What illness was it? ____

7.3 Did you have diarrhea (at least three episodes of liquid or loose stools in one day) during the past two weeks? ☐ Yes ☐ No

8 How many days of work have you missed because of illness in the past year (past 12 months)? ______ days

8.1 If you do not have an official employment, how many days have you been unable to attend to your daily chores in the past year (past 12 months)? _____ days

8.2 What illness(es) was it? ____

9 Where do you usually get your drinking water?

☐ Tap water ☐ Open well ☐ Traditional well

☐ Drilled well ☐ Spring ☐ River / pool ☐ Other (specify) _____________

10 Do you boil your drinking water?

☐ Always ☐ Almost always

☐ Sometimes ☐ Never

11 Do you eat pork meat? ☐ Yes ☐ No [*Skip to Q11.a then Q13*]

11.a If no, did you use to eat pork meat? ☐ Yes ☐ No

11.1 How often do you eat pork?

☐ At least once a month ☐ Less than once a month but at least once a year

☐ Less than once a year

12.1 How is the pork that you eat prepared? [*Check all that apply.*]

☐ Boiling ☐ Barbeque

☐ Fried ☐ Others [*Specify*]_______________________________

12.2 Have you ever eaten [*Check all that apply.*]

☐ Raw pork meat ☐ Rare pork meat

☐ Medium cooked pork meat ☐ Well done pork meat

☐ Cannot remember, do not know

12.3 Where do you usually eat pork meat [*Check all that applies*]

☐ At home ☐ At another concession in the village

☐ At the village's market ☐ At another village's market

☐ Other (specify) ____

13 Do use a latrine?

☐1 Yes ☐2 No [*Skip to Q14*]

13.1 How often do you use a toilet when you have to defecate?

☐1 Always ☐2 Sometimes ☐3 Never

15 Have you ever owned pigs (now or in the past)? *[If they answer "yes", read options 1, 2 and 3]*

☐1 Yes, in the past 12 months ☐2 Yes, one (1) to five (5) years ago

☐3 Yes, more than five (5) years ago

☐4 No [*Skip to Q 17*]

16 Were you ever told that your pigs or piglets were infected with cysts (cysticercosis)?

☐1 Yes ☐2 No [*Skip to Q 17*]

16.1. When were you told that your pig or piglets were infected with cysts (cysticercosis)?

☐1 In the past year ☐2 One (1) to five (5) years ago

☐3 More than five (5) years ago

☐4 Never told (skip to Q 17) ☐5 Can not remember, do not know (Skip to Q 17)

17 Have you ever seen or heard of white nodules (rice) in pig carcasses?

☐1 Yes ☐2 No [*Skip to Q 18*]

17.1 Where can you find nodules on a live pig?

☐1 It is not possible to find them on a live pig

☐2 Under the skin ☐3 Under the tongue

☐4 I don't know ☐5 Somewhere else [*Specify*] _______________

17.2 How do pigs get these nodules?

☐1 By eating human faeces ☐2 By eating pig faeces

☐3 From another infected pig ☐4 Other [*Specify*] ____________________

☐5 I don't know

17.3 How did you hear about those nodules in pigs?

☐1 By a meat inspector ☐2 By a pig trader

☐3 BY a traditional healer ☐4 At the radio / in the newspaper

☐5 By a friend ☐6 By ÉFÉCAB

☐6 Other (spécify) ____

18 Have you ever heard of tapeworm infection in humans?

☐1 Yes ☐2 No [*Skip to Q 19*]

18.1 How did you learn about it?

☐1 By a doctor ☐2 By a friend or family member

☐3 By a traditional healer ☐4 On the radio / newspaper

☐5 Other [*Specify*] ____

18.2 How does a person know if they have a tapeworm?

☐1 They can see it in their faeces ☐2 They have diarrhoea

☐3 They have fever ☐4 Other [*Specify*] _________________________

☐5 I don't know

18.3 Have you ever had a tapeworm or seen small parts (segments) of worms that look like rice grains in your faeces? (*Show photographs of proglottids*)

☐1 Yes ☐2 No [*Skip to Q 18.4*]

☐3 I don't know/can not remember [*Skip to Q 18.4*]

18.3.1 When that happened, what did you do? [*check all that applies*]

☐1 Went to a primary health care provider (hospital, clinic, dispensary)

☐2 Went to the pharmacy to get a drug to treat it

☐3 Went to a traditional healer ☐4 Did nothing

☐5 I can not remember, I do not know

18.4 How does a person get tapeworm infection?

☐1 They do not wash their hands ☐2 They eat undercooked pig meat

☐3 They are in contact with an infected person ☐4 Other [*Specify*] _________

☐5 I don't know

19 Have you ever had skin nodules or hard lumps under the skin? [*Show photograph of person with subcutaneous cysticercosis nodules*]

☐1 Yes, currently has ☐2 Yes in the past year, but not currently

☐3 Yes, one year or more ago, but not currently ☐4 No

☐5 Can not remember, do not know

20 Have you ever had bad headaches that did not go away and that got worse over time?

☐ Yes, currently has ☐ Yes in the past year, but not currently

☐ Yes, one year or more ago, but not currently ☐ No [*Skip to Q21*]

☐ Cannot remember, do not know [*Skip to Q21*]

20.1 Were these headaches bad enough to keep you from doing your daily chores, work or going to school?

☐ Yes ☐ No ☐ Can not remember, do not know

[***If any 'yes' to question 20 and 'yes' or 'can't remember/don't know' to question 20.1 – NOTE ON PDA that this person should be examined by the field doctor***]

20.2 How old were you when this type of headaches first happened?

☐1 I was a child (less than 15) and I was _____________________ years old

☐2 I was a young adult (15-19) and I was ____________________ years old

☐3 I was an adult and I was (20 or more) and I was _____________ years old

☐4 Cannot remember, do not know

20.3 When you have headaches, do you have any trouble with your vision, such as black spots, or seeing zig-zag or wavy lines or numbness in your fingers, arms or legs?

☐ Yes ☐ No ☐ Cannot remember, do not know

20.4 When you have headaches, do you also suffer from nausea or vomiting?

☐ Yes ☐ No ☐ Cannot remember, do not know

21 Have you ever had any of the following?

21.1 Sudden loss of consciousness and episodes of incontinence or foaming of the mouth or tongue biting?

☐1 Yes, currently has ☐2 Yes in the past year, but not currently

☐3 Yes, one year or more ago, but not currently

☐4 No [*Skip to Q 21.2*] ☐5 Cannot remember, do not know

[***If options 1,2, or 3 -- NOTE ON THE PDA that this person must be examined by the team doctor***]

21.1.1 (If yes) How often has this happened?

☐1 Only once ☐2 More than once

21.1.2 How old were you when this first happened?

☐1 I was a child (less than 15) and I was _____________________ years old

☐2 I was a young adult (15-19) and I was ____________________ years old

☐3 I was an adult and I was (20 or more) and I was _____________ years old

☐4 Can not remember, do not know

21.2.3 When did it happen for the first time?

☐1 In the past year (past 12 months) ☐2 From 1 to 2 years ago

☐3 From 3 to 4 years ago ☐4 At least 5 years ago

☐5 Can not remember, do not know

21.2 A brief period of absence(s) or loss(es) of contact with the surroundings that starts suddenly?

☐1 Yes, currently has ☐2 Yes in the past year, but not currently

☐3 Yes, one year or more ago, but not currently

☐4 No [*Skip to Q 21.3*] ☐5 Can not remember, do not know (Skip to Q 21.3)

[***If options 1,2, or 3 -- NOTE ON THE PDA that this person must be examined by the team doctor***]

21.2.1 How often has this happened?

☐1 Only once ☐2 More than once

21.2.2 How old were you when this first happened?

☐ I was a child (less than 15 years old) and I was__________ years old

☐ I was a young adult (15-19 years old) and I was__________ years old

☐ I was an adult (more than 20 years old) and I was__________ years old

☐ Can not remember, do not know

21.2.3 When did it occur for the first time?

☐ During the past year (past 12 months) ☐ From 1 to 2 years ago

☐ From 3 to 4 years ago ☐ At least 5 years ago

☐ Can not remember, do not know

21.3 Uncontrollable twitching or jerking or abnormal movements of one or more limb(s) (convulsions) that starts suddenly and lasts for a period of a few minutes?

☐1 Yes, currently has ☐2 Yes in the past year, but not currently

☐3 Yes, one year or more ago, but not currently

☐4 No [*Skip to Q 21.4*] ☐5 Can not remember, do not know [*Skip to Q 21.4*]

[***If options 1,2, or 3 -- NOTE ON THE PDA that this person must be examined by the team doctor***]

21.3.1 How often has this happened?

☐1 Only once ☐2 More than once

21.3.2 How old were you when this first happened?

☐ I was a child (less than 15 years old) and I was__________ years old

☐ I was a young adult (15-19 years old) and I was__________ years old

☐ I was an adult (more than 20 years old) and I was__________ years old

☐ Can not remember, do not know

21.3.3 When did it occur for the first time?

☐ During the past year (past 12 months) ☐ From 1 to 2 years ago

☐ From 3 to 4 years ago ☐ At least 5 years ago

☐ Can not remember, do not know

21.4 Sudden onset of a brief period of hearing or smelling or seeing things that are not there or feeling strange body sensations?

☐1 Yes, currently has ☐2 Yes in the past year, but not currently

☐3 Yes, one year or more ago, but not currently

☐4 No [*Skip to Q 21.5*] ☐5 Can not remember, do not know [*Skip to Q 21.5*]

[***If options 1,2, or 3 -- NOTE ON THE PDA that this person must be examined by the team doctor***]

21.4.1 How often has this happened?

☐1 Only once ☐2 More than once

21.4.2 How old were you when this first happened?

☐ I was a child (less than 15 years old) and I was__________ years old

☐ I was a young adult (15-19 years old) and I was__________ years old

☐ I was an adult (more than 20 years old) and I was__________ years old

☐ Can not remember, do not know

21.4.3 When did it occur for the first time?

☐ During the past year (past 12 months) ☐ From 1 to 2 years ago

☐ From 3 to 4 years ago ☐ At least 5 years ago

☐ Can not remember, do not know

21.5 Were you ever told that you had epilepsy or that you had had an epileptic seizure?

☐1 Yes, currently has ☐2 Yes in the past year, but not currently

☐3 Yes, one year or more ago, but not currently ☐4 No

☐5 Can not remember, do not know

[***If options 1,2, or 3 -- NOTE ON THE PDA that this person must be examined by the team doctor***]

21.5.2 How old were you when this first happened?

☐ I was a child (less than 15 years old) and I was__________ years old

☐ I was a young adult (15-19 years old) and I was__________ years old

☐ I was an adult (more than 20 years old) and I was__________ years old

☐ Can not remember, do not know

21.5.3 When were you told you had epileptic seizures or epilepsy for the first time?

☐ During the past year (past 12 months) ☐ From 1 to 2 years ago

☐ From 3 to 4 years ago ☐ At least 5 years ago

☐ Can not remember, do not know

21.6 Have you ever had seizures or fits?

☐1 Yes, currently has ☐2 Yes in the past year, but not currently

☐3 Yes, one year or more ago, but not currently

☐4 No [*Skip to Q 22*] ☐5 Can not remember, do not know [*Skip to Q 22*]

21.6.1 How often has this happened?

☐1 Only once ☐2 More than once

[***If options 1,2, or 3 -- NOTE ON THE PDA that this person must be examined by the team doctor***]

21.6.2 How old were you when this first happened?

☐ I was a child (less than 15 years old) and I was__________ years old

☐ I was a young adult (15-19 years old) and I was__________ years old

☐ I was an adult (more than 20 years old) and I was__________ years old

☐ Can not remember, do not know

21.6.3 When did it occur for the first time?

☐ During the past year (past 12 months) ☐ From 1 to 2 years ago

☐ From 3 to 4 years ago ☐ At least 5 years ago

☐ Can not remember, do not know

[If the interviewee has answered "no" to questions 20 to 21.6, the interview is finished. Go to last page and complete questions 30 & 31 based on observation.]

THANK YOU VERY MUCH FOR YOUR COOPERATION

[Otherwise, please continue with the questionnaire]

22 Have you had any of the following?

22.1 Head injury that made you lose consciousness? ☐ 1Yes ☐2 No [*Skip to Q 22.2*]

22.1.1 If yes, when did your seizure symptoms or headaches start?

☐1 Before head injury ☐2 Soon after head injury

☐3 Long time after the head injury ☐4 Can not remember, do not know

22.2 Meningitis (brain infection) during childhood? ☐1 Yes ☐2 No

22.2.1 If yes, when did your seizure symptoms or headaches start?

☐1 Before meningitis ☐2 Soon after meningitis

☐3 Long time after meningitis ☐4 Can not remember, do not know

23 What happens to you when you have a seizure or a fit? _____________________________

____

____

23.1 What happens to you when you have a headache? _____________________________

____

____

24 Have you ever hurt yourself when you lose consciousness or during a seizure?

☐1 Yes ☐2 No

☐3 I do not lose consciousness or have seizures [*Skip to Q 25*]

☐4 Can not remember [*Skip to Q 25*]

24.1 If yes, how did you hurt yourself?

☐1 Fell in the fire ☐2 Fell in the water

☐3 Fell off your bicycle ☐4 Fell while walking along the road

☐5 Cut yourself ☐6 Other [*Specify*] ________________________

(Interviewer: Read the following statement)

Now I want to ask you a few questions about your treatments for *[insert name of symptom or condition they reported having in questions 20 to 21.6. Note that there may be more than one condition that applies]*

26 Have you ever consulted a health provider because of this condition?

☐2 No [*Skip to Q 27*] ☐3 Can not remember [*Skip to Q 27*]

☐1 Yes

26.2 When was the last time you consulted a health provider for your condition?

☐1 Within the past month ☐2 Within the past year

☐3 From one (1) to five (5) years ago ☐4 More than five (5) years ago

☐5 Can not remember, not sure

26.3 What kind of health provider(s) did you consult and how many times in the past 5 years [*check several boxes if appropriate*]?

☐ A health professional /_______ times ☐2 A traditional healer/_________times

☐ Other (specify ____________________________________)/________ times

☐ Can not remember, not sure

27 Have you ever been hospitalised because of this condition?

☐ No [*Skip to Q 28*] ☐ Can not remember [*Skip to Q 28*]

☐ Yes

27.1 When you were last hospitalized, did someone come with you?

☐ Yes ☐ No [*Skip to Q 27.2*] ☐ Can not remember [*Skip to Q 27.2*]

27.1.1 Who came with you

☐1 Mother ☐2 Father

☐3 Brother/sister ☐4 Children [*indicate how many*] ________

☐5 Others (specify) ____

27.2 How many times have you been hospitalised in the past 5 years? _________times

27.3 When were you <u>last</u> hospitalised? ________________(months)

27.3.1 How many days did you stay in hospital? ____________________ (days)

28. Did you ever have any medical tests because of this condition?

☐2 No [*Skip to Q 29*] ☐3 Cannot remember, do not know [*Skip to Q 29*]

☐1 Yes

28.2 What kind of test was it (check as many boxes as appropriate)?

☐1 Blood test ☐2 CT scan of the brain

☐3 X-Ray ☐6 Stool examination

☐8 Spit test

☐5 Other [*specify*] _____________________

☐7 Can not remember, not sure

28.3 When was the last time you had a medical test for this condition?

☐1 Within the past month ☐2 Within the past year

☐3 From one (1) to five (5) years ago ☐4 More than five (5) years ago

☐5 Can not remember, not sure

29. Have you ever taken medicine to treat this condition?

☐2 No [*end of interview*] ☐3 Can't remember, do not know [*end of interview*]

☐1 Yes

29.2 When was the last time you used medication for your condition?

☐1 Within the past month ☐2 Within the past year

☐3 From one (1) to five (5) years ago [*end of interview*]

☐4 More than five (5) years ago [*end of interview*]

☐5 Can not remember, not sure [*end of interview*]

29.3 What medication was it and how many times in the past year did you have to use some (check several boxes if appropriate)?

☐1 Carbamazepine/Tegretol______ times ☐2 Phenytoin/Dihydan_________ times

☐3 Valproic acid/Dépakin________ times ☐4 Phenobarbital/Gardénal _______times

☐5 Paracétamol____ times at____ mg each time

☐6 Paracétamol/dextropropoxyphène _______times at ____ mg each time

☐7 Dihydroergotamine (Séglor, Tamik) _______times at ____ mg each time

☐8 Laroxyl _______times at ____ mg each time

☐9 Traditional medicine ________ times

☐10 6 Other (specify _________) ____times

☐11 Can not remember, not sure

THIS IS THE END OF THE INTERVIEW

THANK YOU VERY MUCH FOR YOUR COOPERATION

INTERVIEWER: ____DATE OF INTERVIEW
