# Supplementary material for: Factors Associated with the 18-Month Cumulative Incidence of Seroconversion of Active Infection with Taenia solium Cysticercosis: A Cohort Study among Residents of 60 Villages in Burkina Faso
Source: Am J Trop Med Hyg. 2018 Sep 4;99(4):1018–27. doi: 10.4269/ajtmh.18-0294 (PMC6159582; doi:10.4269/ajtmh.18-0294)
Supplement: Supplementary file 2 [file tpmd180294.SD2.pdf]

ÉFÉ CAB

Improving pig management to prevent epilepsy in Burkina Faso

Centre Hospitalier Universitaire Souro Sanou, AFRICSanté & University of Oklahoma Health Sciences Center

CHIEF OF HOUSEHOLD QUESTIONNAIRE

Village ___________________________________

Concession _______________________________

Household number ________________________

Last Name _____________________________________ First Name ___________________

1. How long have you lived in this village?______ (yrs.)
2. How many people live in your concession? ________ (nb of people)
3. Where does your family usually get its drinking water?

   ☐ Tap water ☐ Open well ☐ Bore-hole well

   ☐ Traditional well ☐ Drilled well ☐ Spring

   ☐ River / pool ☐ Other (specify) ____________________________________
4. Is the household's drinking water boiled?

   ☐ Always ☐ Almost always

   ☐ Sometimes ☐ Never
5. What type of toilet does your household have?

   ☐ Flushable toilet ☐ Traditional latrine with a hole

   ☐ Improved and ventilated latrine ☐ No toilet / bushes / field

   ☐ Other (specify) ____
6. Does your household own [*check all that applies*]?

   ☐ Electricity ☐ A radio ☐ A television

   ☐ A land line phone ☐ A mobile phone ☐ A refrigerator
7. What kind of fuel does your household use for cooking [*check all that applies*]?

   ☐ Electricity ☐ Bottle / natural gas ☐ Biogas

   ☐ Kerosene / oil ☐ Coal ☐ Wood / straw

   ☐ Manure / cattle manure ☐ Other (specify) _____________________________________

8. Does any member of your household own [*check all that applies*]?

☐ A bicycle ☐ A motorcycle or a moped

☐ A car or a truck ☐ A tractor

☐ A plough ☐ A cart

9. Does your household own livestock [*check all that applies*]?

9.1 Cattle ☐ No ☐ Yes (specify how many) ________________________

9.2 Sheep ☐ No ☐ Yes (specify how many) ________________________

9.3 Goats ☐ No ☐ Yes (specify how many) ________________________

9.4 Donkeys ☐ No ☐ Yes (specify how many) ________________________

9.5 Pigs ☐ No ☐ Yes (specify how many) ________________________

9.6 Poultry ☐ No ☐ Yes (specify how many) ________________________

For each member of the family, please tell me their name, how they are related to you, how old they are, whether they are male or female, and whether they have ever had an epileptic seizure. Let's begin with the oldest:

Name	Relationship to you	Age (years)	Sex (M or F)	History of Epilepsy or Seizures (Y or N)	History of severe headaches (Y or N)
