# Supplementary material for: Factors Associated with the 18-Month Cumulative Incidence of Seroconversion of Active Infection with Taenia solium Cysticercosis: A Cohort Study among Residents of 60 Villages in Burkina Faso
Source: Am J Trop Med Hyg. 2018 Sep 4;99(4):1018–27. doi: 10.4269/ajtmh.18-0294 (PMC6159582; doi:10.4269/ajtmh.18-0294)
Supplement: Supplementary file 3 [file tpmd180294.SD3.pdf]

ÉFÉ CAB

Improving pig management to prevent epilepsy in Burkina Faso

Centre Hospitalier Universitaire Souro Sanou, AFRICSanté & University of Oklahoma Health Sciences Center

MOTHER'S QUESTIONNAIRE

Village ______________________________

Concession ___________________________

Household number _____________________

Mother's Last name : ______________________ First Name : __________________________

Role of the "mother"

☐ Mother in a monogamous family ☐ Eldest mother of a polygamous family

☐ Person in charge of cooking ☐ Other (specify) ____________________

10. Do you eat pork meat? ☐ Yes ☐ No [*Go to Q13*]

10.1 How often do you eat pork meat?

☐ At least once a month ☐ Less than once a month but at least once a year

☐ Less than once a year ☐ Never

11. How do you usually prepare the pork?

☐ Boiling ☐ Barbeque

☐ Fried ☐ Others (specify)___________________________

☐ I never prepare pork meat

12. When you prepare pork, how well cooked do you serve it [*check all that applies*]?

☐ Raw ☐ Rare

☐ Medium ☐ Well cooked

☐ No preference ☐ I do not prepare pork meat

13. Do members of your family have access to a latrine?

☐ Yes ☐ No [*Skip to Q 14*]

13.1 How many members of your household use the toilet to defecate? _______

13.2 How often do family members use the latrine to defecate?

☐ Almost always ☐ Sometimes ☐ Never

14. Does someone living in your household keep pigs?

☐ Yes ☐ No [*Skip to Q 15*]

14.1 Who takes care of the pigs? [*Check all that applies*]

☐ Me (mother) ☐ My husband

☐ One of my daughters ☐ One of my sons

☐ All my children ☐ Other (specify) ______________________

15. Has a member of your household ever kept pigs? *[If they answer "yes", ask "How long ago did you own pigs?"]*

☐ No (skip to end of interview) ☐ Yes, in the past year

☐ Yes, one (1) to five (5) years ago ☐ Yes, more than five (5) years ago

15.1. Who took care of the pigs when you had them?

☐ Me (mother) ☐ My husband

☐ One of my daughters ☐ One of my sons

☐ All my children ☐ Other (specify) ______________________

THIS IS THE END OF THE INTERVIEW

THANK YOU VERY MUCH FOR YOUR COOPERATION

INTERVIEWER: ________________________ DATE OF INTERVIEW: ____________

Interviewers, please note the following characteristics regarding the household:

16. What is the floor made of?

☐ Natural floor – earth / sand ☐ Finished floor - cement

☐ Finished floor – Tiles ☐ Finished floor – vinyl / asphalt

☐ Finished floor – carpet ☐ Other (specify) ______________________

17. What is the roof made of?

☐ Straw ☐ Mud ☐ Sheet metal

☐ Concrete ☐ Other (specify) ______________________

18. What are the walls made of?

☐ Earthen bricks ☐ Machine cut bricks ☐ Stone

☐ Fired bricks ☐ Cement or concrete ☐ Metal or sheet metal

☐ Straw ☐ Other (specify) ______________________
