# Supplementary material for: Factors Associated with the 18-Month Cumulative Incidence of Seroconversion of Active Infection with Taenia solium Cysticercosis: A Cohort Study among Residents of 60 Villages in Burkina Faso
Source: Am J Trop Med Hyg. 2018 Sep 4;99(4):1018–27. doi: 10.4269/ajtmh.18-0294 (PMC6159582; doi:10.4269/ajtmh.18-0294)
Supplement: Supplementary file 4 [file tpmd180294.SD4.pdf]

ÉFÉ CAB

Improving pig management to prevent epilepsy in Burkina Faso

Centre Hospitalier Universitaire Souro Sanou, AFRICSanté & University of Oklahoma Health Sciences Center

PIG QUESTIONNAIRE

District ________________________________

Village _________________________________

Hut (house) number ______________________

Last name : ________________________ First Name : ____________________________

1. What is your position in the household?

☐ Mother ☐ Father

☐ One of the daughters ☐ One of the sons

☐ Other [*Specify*]________________________

2. How many pigs do you keep? _________ ☐ Does not know number

2a. What type of pigs do you keep [*indicate number of each type*]

☐ Foreign ________ ☐ Indigenous __________________

☐ Other (specify) __________________________________

2b. Where do you usually buy your pigs and how much do you pay?

☐ I never buy pigs, I use the ones my own sows deliver

☐ From another farmer in my village and I pay _______________________

☐ From a farmer in a neighboring village ________________________ [*indicate village name*] and I pay ______________________________

☐ At the market in another village ________________________ [*indicate village name*] and I pay _____________________________

☐ Other [*Specify*]________________________

3. How many of these pigs are owed by your household ? __________________ [*indicate a number*]

4. Among the pigs owned by your household, how many are kept for [*read each option and indicate the number kept for that reason*]

☐ Home eating ______________ ☐ Sell live animals (not to abattoir) ________

☐ Sell the meat to someone else ________ ☐ Sell the pig to the abattoir ____________

☐ Reproduction [*Go to 4.1*]______________ ☐ Other [*Specify*]____________________

4.1 How many piglets did the last sow that gave birth have? _________

4.2 How many times did you need to take her to the boar before she got pregnant? __________

4.3 How many litters in the past 12 months did the last sow that deliver have? _______________

5. How do you keep your pigs? [*read questions 5.1 to 5.2 one by one*]

5.1 During the rainy season

☐ In a pen ☐ Free range

☐ Tethering (tied up?) ☐ Other [*Specify*]_______________

5.2 During the dry season

☐ In a pen ☐ Free range

☐ Tethering ☐ Other [*Specify*]_______________

6. What do your pigs eat? [*check all that apply*]

☐ Pasture ☐ Slop (from dolo or beer)

☐ Kitchen leftovers ☐ Commercial feeds

☐ Other [*Specify*]____________________

7. How often do you slaughter pigs at home?

☐ Never [*Skip to Q 8*] ☐ Cannot remember, do not know [*Skip to Q8*]

☐ At least once a month ☐ Less than once a month but at least once a year

☐ Less than once a year

7.1 If ever, how often was the meat inspected by a meat inspector?

☐ Always ☐ Almost always

☐ Sometimes ☐ Never

☐ Cannot remember, do not know

8. At what price do you usually sell your pigs when they are ready to be slaughtered [*specify the usual weight, currency used, this can be money or barter*]?

____

9. At what price do you usually sell your piglets around weaning (aged 4 months or less) [*specify the currency used, this can be money or barter*]? ______________________________________

10. For what do you use the profits from selling your pigs ? *[check all that apply]*

☐ To send children to school ☐ To buy food

☐ To invest in a business ☐ For savings

☐ To treat a health problem

☐ Other [*Specify*] ____

11. Have you ever seen or heard of white nodules (rice) in pig carcasses?

☐ Yes ☐ No [*Interview is over*]

11.1 Where can you find nodules on a live pig?

☐ It is not possible to find them on a live pig

☐ Under the skin ☐ Under the tongue

☐ I don't know ☐ Somewhere else [*Specify*]______________

11.2 How do pigs get these nodules?

☐ By eating human faeces ☐ By eating pig faeces

☐ From another infected pig ☐ Other [*Specify*] ____________________

☐ I don't know

11.3 What would you do if you discovered that your pig had these nodules?

☐ Sell the pig ☐ Treat it with herbs

☐ Pierce the nodules ☐ Other [*Specify*] ______________________

☐ I don't know

12. At what price would you be able to sell pigs that have nodules [*specify the usual weight, currency used, this can be money or barter*]? ____

13. At what price would you be able to sell piglets around weaning if they have nodules (aged 4 months or less) [*specify the currency used, this can be money or barter*]?

____

THIS IS THE END OF THE INTERVIEW

THANK YOU VERY MUCH FOR YOUR HELP

14. [*Interviewer, indicate how many adult pigs were kept in each way listed below during the interview]*

☐ In a pen _____________ ☐ Free range _______________

☐ Tethered ______________ ☐ Other [*Specify*]_______________

15. [*Interviewer, indicate how many adult piglets were kept in each way listed below during the interview]*

☐ In a pen _____________ ☐ Free range _______________

☐ Tethered ______________ ☐ Other [*Specify*]_______________

16. Indicate the gender of the pig sampled for a blood sample ____________________

17. Indicate the gender of the pig sampled for a blood sample ___________________

18. Indicate the approximate age of the pig sample for a blood sample _____________

INTERVIEWER: ____________________________DATE OF INTERVIEW:_____________
