# Supplementary material for: Factors Associated with the 18-Month Cumulative Incidence of Seroconversion of Active Infection with Taenia solium Cysticercosis: A Cohort Study among Residents of 60 Villages in Burkina Faso
Source: Am J Trop Med Hyg. 2018 Sep 4;99(4):1018–27. doi: 10.4269/ajtmh.18-0294 (PMC6159582; doi:10.4269/ajtmh.18-0294)
Supplement: Supplementary file 5 [file tpmd180294.SD5.pdf]

ÉFÉ CAB

Improving pig management to prevent epilepsy in Burkina Faso

Centre Hospitalier Universitaire Souro Sanou, AFRICSanté & University of Oklahoma Health Sciences Center

FOLLOW-UP SCREENING QUESTIONNAIRE

Last name : ________________________ First name : ______________________________

Questionnaire number _____________________

ID number |__|__|__|__|__|__|__|__|__|__|__|__|

Village ___________________________________

Concession _______________________________

Household number ________________________

1. Have you changed employment in the past 12 months? ☐ Yes ☐ No [*Go to Q2*]

1.1 What is your new occupation? [*housewife is an occupation*]

☐ Farmer ☐ Small business ☐ Handicraft

☐ Salaried (specify) _______________ ☐ Housewife

☐ Other (specify) _____________________

1.1.1 What is your new monthly salary? ____________________ CFA

2. How many days of work have you missed because of illness in the past month? ______ days

2.1 If you do not have an official employment, how many days have you been unable to attend to your daily chores or missed school in the past month? _____ days

2.2 What illness was it? ____

3. How many days of work have you missed because of illness in the past year (past 12 months)? ______ days

3.1 If you do not have an official employment, how many days have you been unable to attend to your daily chores in the past year (past 12 months)? _____ days

3.2 What illness was it? ____

4. Have you eaten pork meat in the past 12 months? ☐ Yes ☐ No [*Skip to Q7*]

4.1 How many times have you eaten pork meat in the past 12 months?

☐1 Daily (every day) ☐2 One to six times per week

☐3 Less than once per week but more than once per month

☐4 Less than once per month

☐5 I do not know, I do not remember

4.2 During the past 12 months, did you eat pork meat cooked the following way? [*Cochez toutes les réponses qui s'appliquent*]

☐1 Raw pork meat ☐2 Rare pork meat

☐3 Medium cooked pork meat ☐4 Well done pork meat

☐5 Cannot remember, do not know

4.3 Where did you eat pork meat in the past 12 months [*Check all that applies*]

☐ At home ☐ At another concession in the village

☐ At the village's market ☐ At another village's market

☐ Other (specify) ____

☐ Cannot remember, do not know

5. Did you use the latrine in the past 12 months?

☐1 Yes ☐2 No [*Skip to Q8*]

5.1 How often did you use a toilet when you had to defecate in the past 12 months?

☐1 Always ☐2 Sometimes ☐3 Never

6. Do you keep pigs now?

☐ Yes ☐ No

7. Have you ever seen or heard of white nodules (rice) in pig carcasses?

☐1 Yes ☐2 No [*Skip to Q 8*]

7.1 Where can you find nodules on a live pig?

☐1 It is not possible to find them on a live pig

☐2 Under the skin ☐3 Under the tongue

☐4 I don't know ☐5 Somewhere else [*Specify*] _____________

7.2 How do pigs get these nodules?

☐1 By eating human faeces ☐2 By eating pig faeces

☐3 From another infected pig ☐4 Other [*Specify*] __________________

☐5 I don't know

7.3 How did you hear about those nodules in pigs?

☐1 By a meat inspector ☐2 By a pig trader

☐3 BY a traditional healer ☐4 At the radio / in the newspaper

☐5 By a friend ☐6 By ÉFÉCAB

☐6 Other (spécify) ____

8 Have you ever heard of tapeworm infection in humans?

☐1 Yes ☐2 No [*Skip to Q 9*]

8.1 How did you learn about it?

☐ By a doctor ☐ By a friend or family member

☐ By a traditional healer ☐ On the radio / newspaper

☐ By ÉFÉCAB

☐ Other [*Specify*] ____

8.2 How does a person know if they have a tapeworm?

☐1 They can see it in their faeces ☐2 They have diarrhea

☐3 They have fever ☐4 Other [*Specify*] _________________________

☐5 I don't know

8.3 Have you had a tapeworm or seen small parts (segments) of worms that look like rice grains in your faeces during the past 12 months? (*Show photographs of proglottids*)

☐1 Yes ☐2 No [*Skip to Q 8.4*]

☐3 I don't know/can not remember [*Skip to Q 8.4*]

8.3.1 When that happened, what did you do? [*check all that applies*]

☐1 Went to a primary health care provider (hospital, clinic, dispensary)

☐2 Went to the pharmacy to get a drug to treat it

☐3 Went to a traditional healer ☐4 Did nothing

☐5 I can not remember, I do not know

8.4 How does a person get tapeworm infection?

☐1 They do not wash their hands ☐2 They eat undercooked pig meat

☐3 They are in contact with an infected person ☐4 Other [*Specify*] __________

☐5 I don't know

9 Have you ever had bad headaches that did not go away and that got worse over time?

☐ Yes, currently has ☐ Yes in the past year, but not currently

☐ Yes, one year or more ago, but not currently ☐ No [*Skip to Q10*]

☐ Cannot remember, do not know [*Skip to Q10*]

9.1 Were these headaches bad enough to keep you from doing your daily chores, work or going to school?

☐ Yes ☐ No ☐ Can not remember, do not know

[***If any 'yes' to question 9 and 'yes' or 'can't remember/don't know' to question 9.1 – NOTE ON PDA that this person should be examined by the field doctor***]

9.2 When you have headaches, do you have any trouble with your vision, such as black spots, or seeing zig-zag or wavy lines or numbness in your fingers, arms or legs?

☐ Yes ☐ No ☐ Cannot remember, do not know

9.3 When you have headaches, do you suffer from nausea or vomiting?

☐ Yes ☐ No ☐ Cannot remember, do not know

10. In the past 12 months, have you had any of the following?

10.1 Sudden loss of consciousness and episodes of incontinence or foaming of the mouth or tongue biting?

☐ Yes, I have them now ☐ Yes, in the past 12 months but not now

☐ No [*Skip to Q10.2*] ☐ Can not remember, do not know [*Skip to Q10.2*]

[***If options 1,2, or 3 -- NOTE ON THE PDA that this person must be examined by the team doctor***]

10.1.1 (If yes) How often has this happened?

☐1 Only once ☐2 More than once

10.2 A brief period of absence(s) or loss(es) of contact with the surroundings that starts suddenly?

☐ Yes, I have them now ☐ Yes, in the past 12 months but not now

☐ No [*Skip to Q10.3*] ☐ Can not remember, do not know [*Skip to Q10.3*]

[***If options 1,2, or 3 -- NOTE ON THE PDA that this person must be examined by the team doctor***]

10.2.1 How often has this happened?

☐1 Only once ☐2 More than once

10.3 Uncontrollable twitching or jerking or abnormal movements of one or more limb(s) (convulsions) that starts suddenly and lasts for a period of a few minutes?

☐ Yes, I have them now ☐ Yes, in the past 12 months but not now

☐ No [*Skip to Q10.4*] ☐ Can not remember, do not know [*Skip to Q10.4*]

[***If options 1,2, or 3 -- NOTE ON THE PDA that this person must be examined by the team doctor***]

10.3.1 How often has this happened?

☐1 Only once ☐2 More than once

10.4 Sudden onset of a brief period of hearing or smelling or seeing things that are not there or feeling strange body sensations?

☐ Yes, I have them now ☐ Yes, in the past 12 months but not now

☐ No [*Skip to Q10.5*] ☐ Can not remember, do not know [*Skip to Q10.5*]

[***If options 1,2, or 3 -- NOTE ON THE PDA that this person must be examined by the team doctor***]

10.4.1 How often has this happened?

☐1 Only once ☐2 More than once

10.5 Were you told that you had epilepsy or that you had had an epileptic seizure?

☐ Yes ☐ No [*Skip to Q 12.3*] ☐ Cannot remember, do not know

[***If options 1,2, or 3 -- NOTE ON THE PDA that this person must be examined by the team doctor***]

10.6 Have you ever had seizures or fits?

☐ Yes, I have them now ☐ Yes, in the past 12 months but not now

☐ No [*end of interview*]

☐ Can not remember, do not know [*end of interview*]

[***If options 1,2, or 3 -- NOTE ON THE PDA that this person must be examined by the team doctor***]

10.6.1 How often has this happened?

☐1 Only once ☐2 More than once

THIS IS THE END OF THE INTERVIEW

THANK YOU VERY MUCH FOR YOUR COOPERATION

INTERVIEWER: ____DATE OF INTERVIEW
